# Delayed Complete Clearance of Recalcitrant Warts Due to Intralesional Measles, Mumps, and Rubella: Case Report and Review

**DOI:** 10.7759/cureus.67225

**Published:** 2024-08-19

**Authors:** Angel Varghese, Nithin M George, Shivani Wadhwa

**Affiliations:** 1 Department of Dermatology, Fakeeh University Hospital, Dubai, ARE; 2 Department of Dermatology, West China School of Medicine, Sichuan University, Sichuan, CHN

**Keywords:** flat warts, common warts, human papillomavirus, periungual warts, recalcitrant warts, mmr for warts, immunotherapy, palmoplantar warts

## Abstract

Warts are a prevalent skin condition that can affect people of any age. They are caused by the human papillomavirus (HPV), a double-stranded DNA virus that can cause benign and malignant lesions and remains latent in the host cells, leading to recurrences. Although warts are benign and spontaneous clearance has been reported over the years, they often cause disfigurement, tend to koebnerize, and can be transmitted to others, making adequate and timely treatment important. Several conventional treatments are available, but none works consistently for all patients. Incomplete responses or recurrences are often bothersome to both patients and dermatologists. Moreover, these treatments are often painful, time-consuming, and can cause significant scarring. Immunotherapy, as an alternative, has found a significant place in the treatment of warts because of its non-destructive action, ease of use, and promising results.

This paper will discuss a healthy 36-year-old Bosnian male with chronic palmoplantar and periungual warts. Despite undergoing multiple destructive and topical treatments, including electrocautery, cryotherapy, carbon dioxide laser, salicylic acid, glycolic acid, 5-fluorouracil, and imiquimod, he could not achieve significant improvement in his skin condition. Subsequent treatment with the intralesional measles, mumps, rubella (MMR) vaccine also showed little improvement during treatment. However, three months without further treatment, the patient reported complete resolution of the warts. Follow-up confirmed the clearance with no recurrence and minor post-inflammatory hypopigmentation. Our patient's delayed response to the MMR vaccine aligns with findings from other studies indicating that the body's immune response may take time to manifest fully.

## Introduction

Warts are a widespread condition that affects individuals of all ages and genders, caused by the human papillomavirus (HPV). The virus can remain dormant in host cells for extended periods, leading to recurrent lesions that can be either benign or malignant [1]. Different HPV strains infect specific body areas, resulting in various wart types: Plantar warts are commonly caused by HPV types 1, 2, 4, 27, and 57 [2].

HPV can be transmitted directly through contact or contaminated objects like towels or razors. Although 65-78% of warts resolve spontaneously within approximately two years, patients often seek treatment due to pain and cosmetic concerns [3]. Treatment options include topical agents such as trichloroacetic acid, salicylic acid, podophyllotoxin, and 5-fluorouracil, as well as destructive methods like radio cautery, cryotherapy, surgical excision, and carbon dioxide laser, which can result in scarring. Systemic treatments, including levamisole and zinc sulfate, have also been tried, but none guarantee complete resolution without recurrence [1].

Unlike destructive therapies that target warts from the outside in, immunotherapy works by enabling the body’s immune system to identify HPV as abnormal and combat it from within. Immunotherapy is defined as a type of biological therapy that employs various substances to either stimulate or suppress the immune system, thereby aiding the body in fighting infections or cancers [4].

## Case presentation

An immunocompetent 36-year-old Bosnian male presented to the Derma Surge department of Fakeeh University Hospital, with several years of history of raised spots on his hand. He had undergone multiple destructive therapies before visiting us, but the lesions had been persistent. Clinical examination revealed multiple verrucous papules and small plaques on the dorsal and ventral aspects of his hands and feet, with a few periungual lesions. A clinical diagnosis of chronic palmoplantar warts was made. We started with conventional destructive therapies. His baseline investigations, including complete blood count, liver, and kidney functions, were all within normal limits. He had no other significant medical history. The patient underwent a total of six sessions of cryotherapy and curettage followed by carbon dioxide laser over three months. He was also prescribed topical keratolytics and imiquimod to use regularly. The patient showed improvement but not complete resolution.

A year later, he presented with a significant worsening of the lesions. Treatment with intralesional measles, mumps, rubella (MMR) was offered, and informed consent was taken. 0.3 ml of MMR vaccine (PRIORIX) was administered in the largest periungual lesion on the right index finger. A total of six sessions were done at intervals of two weeks. At the end of four sessions, there was minimal improvement (less than 50%) in the size and number of lesions. This prompted us to increase the dose, and the next two sessions were done with a dose of 0.5 ml each. On the follow-up, four weeks after the last session, the patient still showed no betterment, so the treatment was stopped. The injected lesion showed a mild reduction in size, not more than 25%.

**Figure 1:**
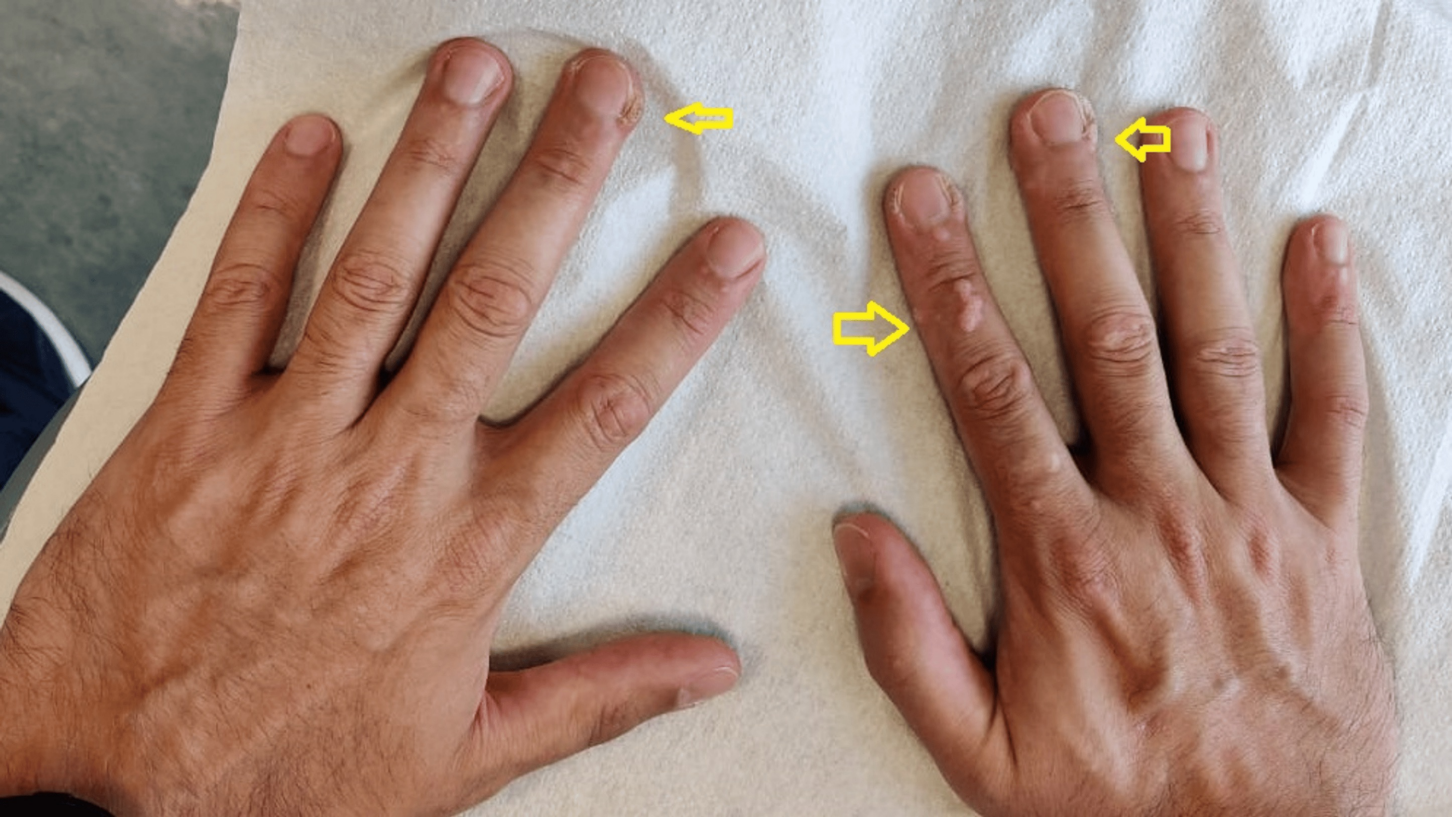
Before treating with MMR injections. The yellow arrows indicate the aggressive lesions on the plantar surface. MMR: Measles, mumps, rubella.

**Figure 2:**
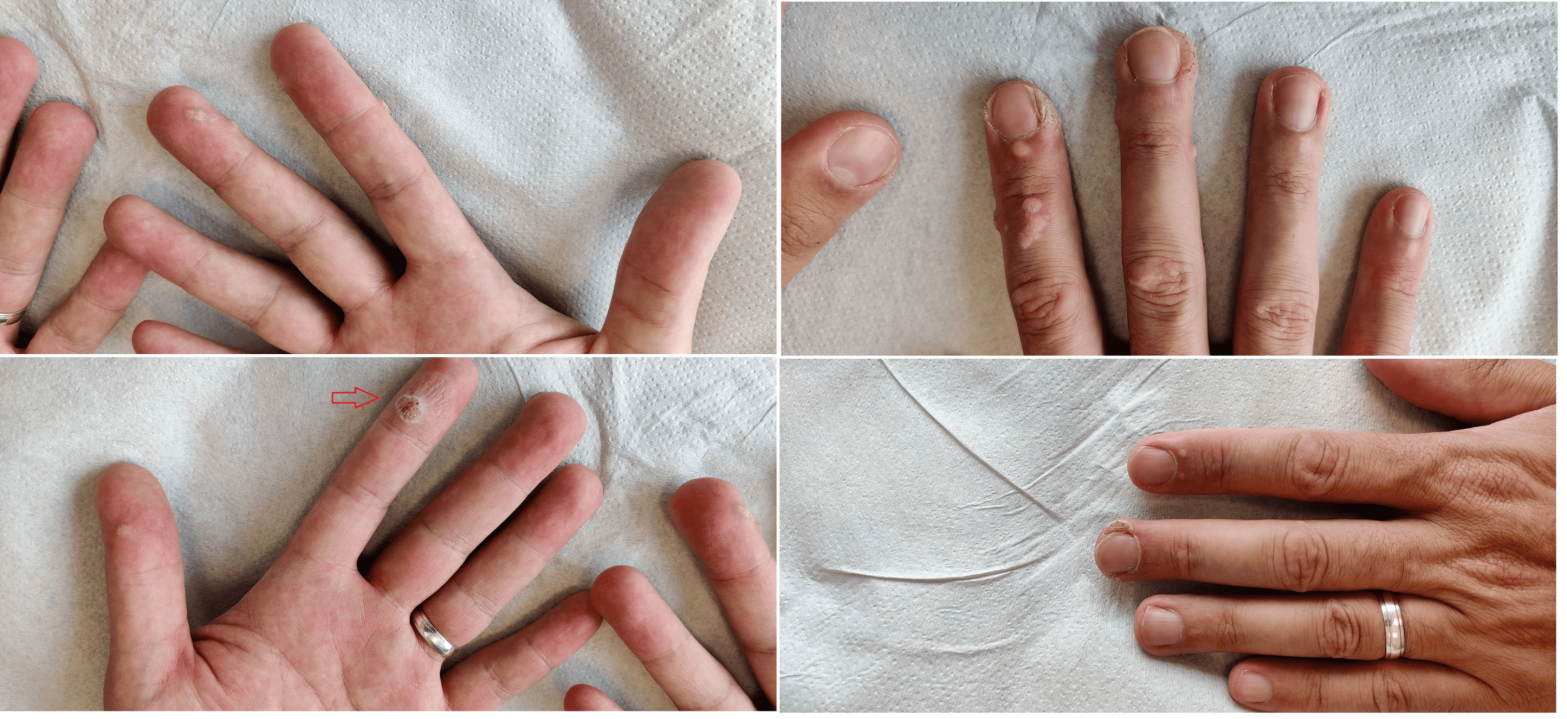
Magnified images of lesions following six sessions of MMR injections. The red arrow denotes an aggressive lesion on the palmar surface of the left index finger. The progress of the lesions was limited compared to their initial state. MMR: Measles, mumps, rubella.

**Figure 3:**
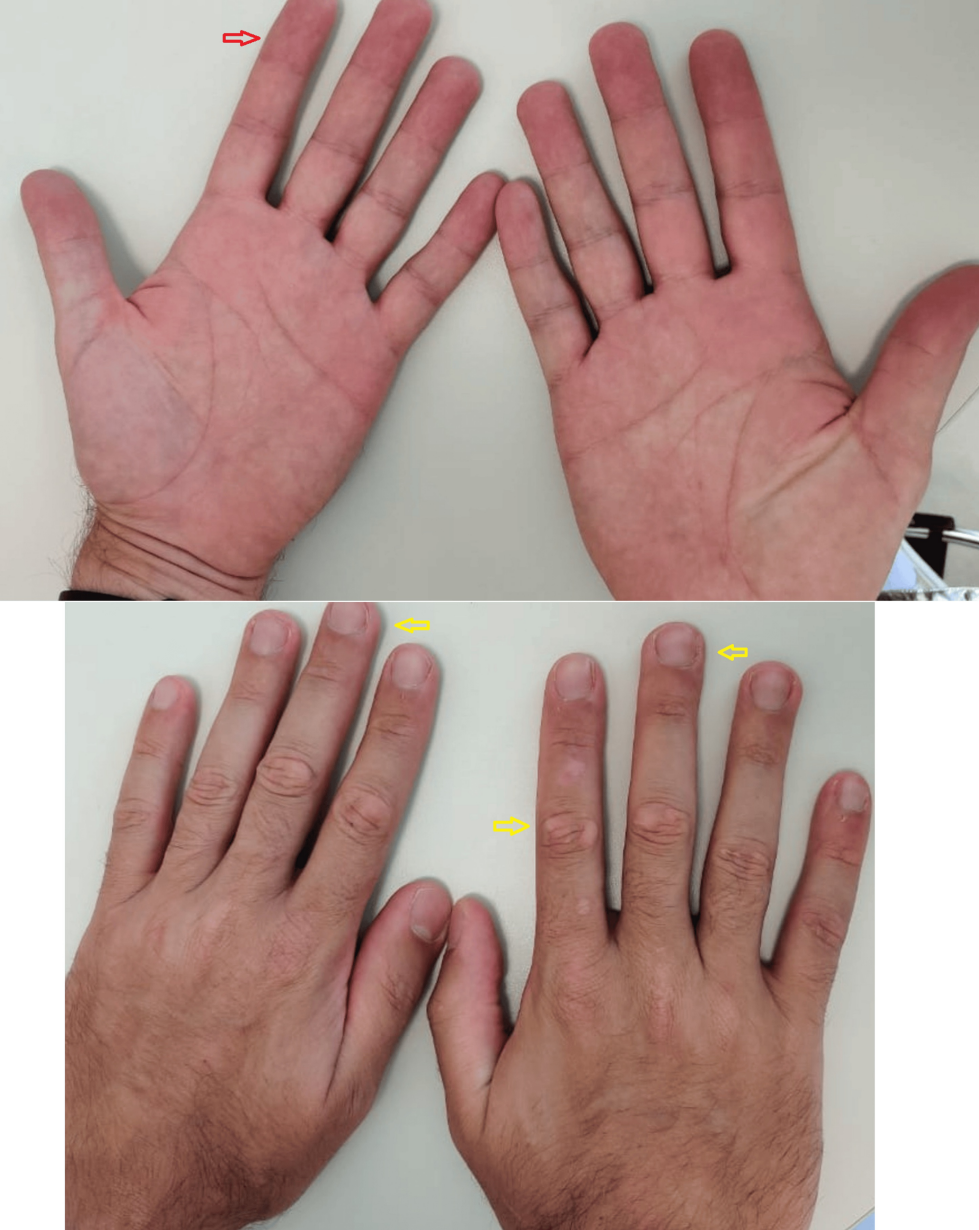
After 32 weeks since the last MMR injections. The red and yellow arrows indicate previously identified aggressive lesions on the palmar and plantar surfaces, respectively, which have now completely resolved. The plantar lesions have left behind mild hypopigmentation. MMR: Measles, mumps, rubella.

**Figure 4:**
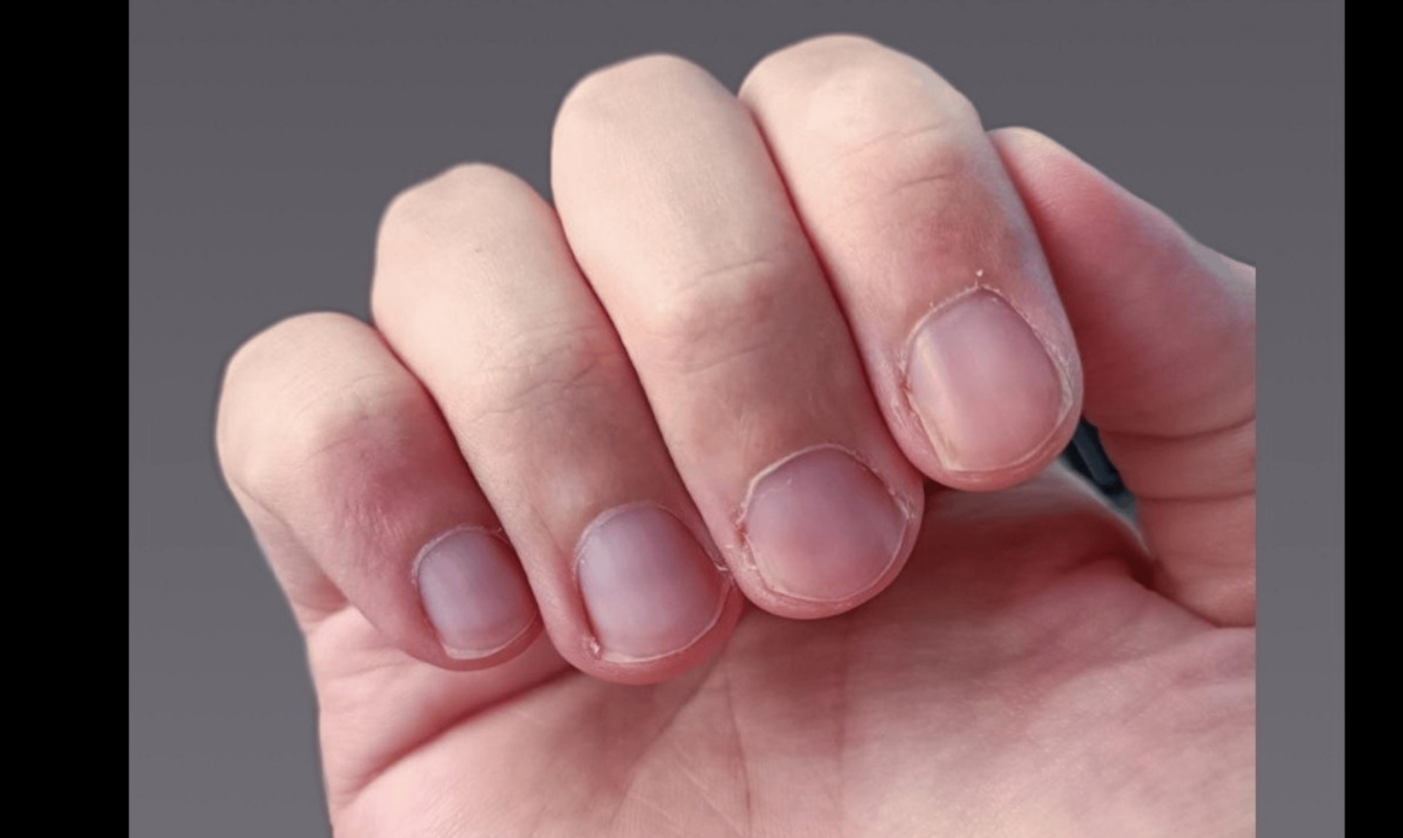
Magnified image of the periungual lesions on the right hand, which have completely resolved, taken 32 weeks after the last injection.

Thirteen weeks after the last session, the patient informed us over the phone that all the lesions had cleared from his hands and feet. He denied any medication use or procedures since the last visit with us. On a follow-up visit, 15 weeks after the last intralesional injection, complete clearance of the injected lesion and other distant lesions was noticed. Post-inflammatory hypopigmentation was noted on some of the lesions, and the patient denies any other side effects after the injections. Another follow-up done at 32 weeks after the last intralesional injection revealed no recurrence, and the patient was very satisfied with the results. Figure 1 shows the plantar and periungual warts that the patient presented with before starting MMR injections. Figure 2 was taken on the follow-up after the sixth injection of MMR, where the lesions showed very minimal improvement. Figure 3 shows the complete clearance of the palmoplantar and periungual lesions, taken after 32 weeks of the last injection of MMR when the patient came for follow-up. Figure 4 is an additional image of the cleared, right periungual lesions taken on the same day, 32 weeks after the last injection. 

## Discussion

Current insights on MMR immunotherapy: what we know

Treating recalcitrant warts presents a persistent challenge for many physicians [1]. Various traditional treatment methods have been employed, but none have proven to be entirely effective, and most are associated with recurrences [3]. Destructive treatments such as electrocautery, lasers, and cryotherapy are often painful and carry a risk of scarring. When used for multiple lesions, these methods can be time-consuming and require numerous visits [1]. Because of these reasons, immunotherapy is becoming increasingly popular, especially in the treatment of refractory warts. Currently, it is being used for recalcitrant warts, recurrent warts, extensive warts, and difficult-to-treat areas such as periungual and palmoplantar sites when traditional methods are exhausted [4].

Based on the route of administration, immunotherapy for recalcitrant warts can be topical, intralesional, or systemic. Intralesional immunotherapy offers several advantages over traditional therapies: it is cost-effective, requires fewer visits for both primary and distant non-injected warts, has fewer side effects, allows for an early return to normal activities, and generally results in minimal scarring [5].

The precise mechanism behind intralesional immunotherapy is not fully understood, but it is believed that the injection triggers the release of various immunoregulatory cytokines such as IL-2, IL-4, IL-5, IL-12, interferon-γ, and tumor necrosis factor-α. This enhances the cell-mediated immune response against HPV, not only at the treated site but also at distant sites. The protection against recurrence can be attributed to long-term vigilance of the immune system against the HPV virus by stimulating persistent memory T cells [6-8]. Table 1 describes the different antigens being used experimentally for warts.

**Table 1:** Various agents used in intralesional immunotherapy for warts. Mw: Mycobacterium indicus pranii; BCG: Bacille Calmette-Guerin; PPD: Purified Protein Derivative; MMR: Measles Mumps Rubella. Different agents used intralesionally as immunotherapy against warts and their respective administration doses [4].

Agents	Administration	Dosage	
Mw Vaccine	Intradermally into multiple lesions/ Intralesional into largest wart	0.1 ml	2-4 weekly, maximum 10 sittings
BCG Vaccine	Intralesional into largest wart	0.1-0.5 ml	2 weeks intervals in 5 sittings
PPD	Intradermal into forearm	0.1 ml	Weekly, for 12 weeks
MMR Vaccine	Single largest wart	0.3-0.5 ml	Fortnightly for up to 5 sittings
Candidal extract	Injected into largest wart	0.1-0.3 ml	3 times a week
Trichophyton	Injected into largest wart	0.3 ml	Every 3 weeks for maximum 5 sittings
Tuberculin	Intralesional into multiple lesions	2.5 units	Every 2 weekly
Vitamin D3	Intralesional into each lesion	0.2 ml of 7.5 mg/ml	2 sittings 4 weeks apart
Interferon alpha	Intralesional into each lesion	1-2 million units	3 days for 3 weeks

We chose MMR as our agent of choice for intralesional immunotherapy because of its demonstrated efficacy, safety profile, ease of availability, and cost-effectiveness. Additionally, using the combined antigens of measles, mumps, and rubella (MMR) may generate a stronger immune response against HPV compared to using a single antigen, positioning MMR as a more effective option among available immunotherapies [1]. A meta-analysis of randomized controlled trials evaluating the effectiveness of various wart treatments, including intralesional immunotherapies, indicated that purified protein derivative (PPD), MMR, and interferon β were more effective than placebo, imiquimod, and cryotherapy in achieving complete resolution at the primary injection site. For distant warts, autoinoculation, PPD, and MMR demonstrated statistically superior results compared to placebo. Notably, MMR also proved more effective than its competitors in reducing recurrence at the treated site in this study [9].

Several studies on the MMR vaccine have reported a diverse range of response rates, which can be attributed to possible confounders such as the demographics of the selected population and their sizes, type and duration of warts, number of injections administered, and the follow-up sessions conducted [10]. Typically, the majority of studies utilize three to six doses of 0.1-0.5 ml, administered at intervals of 2-3 weeks, due to the lack of consensus on the minimum effective dose of the MMR vaccine and the duration of therapy [5]. This is exemplified in the research conducted by Kaur A et al., where they administered three sessions every three weeks, and in the study by Awal G and Kaur S, where five sessions every 2 weeks were used [1,5]. The average number of injections required for complete clearance ranges from 3.7 to 4 in studies by Awal G and Kaur S and Jain SP et al. [1,3].

Awal G and Kaur S [1] demonstrated that having a prior sensitization approach allows for the subjects to be sensitive and have a stronger immune response to the antigen, thus increasing the treatment success rate with MMR; this was achieved by using a 0.1 ml intradermal injection on the volar aspect of the forearm, with a follow-up examination after a week to check for an immune response, indicated by erythema or nodule formation. Research has indicated that increasing the vaccine volume or the number of sessions may improve responses in patients with less favorable outcomes. Following this, we increased the MMR dose from 0.3 ml to 0.5 ml for our patient, who initially displayed a poor response. We also administered the vaccine in two weekly sessions, as this approach may provide better antigenic stimulation.

Response to MMR immunotherapy in follow-up

While many studies have conducted follow-ups from 8 to 24 weeks after the final treatment, a few studies, such as Sobhy MN et al., [11] have followed up with patients up to six months post-treatment, resulting in an 86.9% complete response rate, with no response observed in the control group. Rageh RM et al., [12], a study with shorter follow-ups, demonstrated a clearance rate of 26.7% and no response in 53.3% of cases.

Although our patient did not exhibit significant improvement over several months, a complete resolution was observed, which can be credited to rigorous management and extended follow-ups. This delay may be due to the chronicity and severity of his warts. Prior investigations have produced parallel outcomes, demonstrating that a great number of individuals achieved complete response during follow-up as opposed to immediately after treatment. For instance, Mohtashim M et al. (2018) [6], experimented on 200 subjects with recalcitrant warts where it was found that intralesional MMR injections led to improved effects even after the cessation of treatments. A total of 25.1% of patients showed clearance immediately after five sessions, whereas 37.7% achieved resolution only during the follow-up period. A study conducted by Chauhan PS et al., 2019 [13], reports improved outcomes during the follow-up period after treatment discontinuation in 16 patients out of 42. In this study, 12 patients had successfully achieved complete clearance after four weeks and an additional four patients were able to achieve the same eight weeks following their last MMR injection.

This can be further witnessed in Kaur A et al., which compared the responses between MMR and Mycobacterium indicus pranii (MIP), and observed that continuous improvement in outcome was noted up to 24 weeks post-treatment with their MMR candidates, in contrast to those receiving MIP showing none after 16 weeks [5]. Table 2 compares the response of patients exclusively seen in the follow-up period after the last injection of MMR.

**Table 2:** Percentage of patients attaining complete response in the follow-up period. The table compares the percentage of patients who attained complete responses exclusively in the follow-up periods of their respective studies. Please note the outcome measurement varied in each study; it overall ranged from a 75% to 99% reduction in the size of warts and the return of normal skin markings [5,6,13-16].

Study and Year	Age Mean (SD) in Years	Dose	Number of Sessions	Follow- Up	Percentage of Patients with Complete Response at First Follow-up	Percentage of Patients with Complete Response at Final Follow- up
Kaur A et al. (2021) [5]	29.43	0.5 ml	Every 3 weeks for a maximum of 3 sessions	Every 4 weeks for 24 weeks	After 4 weeks of the last dose 36.67%	20 to 24 weeks after the last dose 76.67%
							Saha A et al. (2022) [14]	34 (10.63)	0.3 ml	Every 2 weeks for a maximum of 3 sessions	Every 4 weeks for 12 weeks	After 4 weeks of the last dose 16.7% (based on reduction of number of lesions)	16 weeks after the last dose 40% (based on number of lesions)
Mohtashim M et al. (2018) [6]	26.26 (8.78)	0.5 ml	Every 2 weeks for a maximum of 5 sessions	At 12 weeks and 24 weeks	After 12 weeks of the last dose 41.4%	24 weeks after the last dose 62.80%
							Zamanian A et al. (2014) [15]	18.9 (12)	0.5 ml	Every 2 weeks for a maximum of 3 sessions	Every 8 weeks for 24 weeks	After 4.2 weeks of the last dose 54.20%	6.4 weeks after the last dose 75%
Chauhan PS et al. (2019) [13]	31.3 (11.15)	0.25 ml	Every 2 weeks for a maximum of 5 sessions	Every 4 weeks for 8 weeks	After 4 weeks of the last dose 48%	8 weeks after the last dose 30.7%
							Rajegowda H et al. (2020) [16]	28.35 (Males) 25.31 (Females)	0.5 ml	Every 3 weeks for a maximum of 3 sessions	Every 4 weeks for 8 weeks	After 3 weeks of the last dose 56.67%	11 weeks after the last dose 63.33%

Zamanian A et al. conducted a double-blind, randomized clinical trial that demonstrated the effectiveness of MMR therapy for warts increases over time [15]. A minimal response to treatment was observed in our patient during the sessions; however, complete clearance without recurrence of warts was accomplished within six months post-discontinuation of treatment. The data presented in Table 2, supported by the cited studies, unequivocally demonstrate that the treatment's positive effects persist for a considerable duration into the follow-up period. It would be prudent to acknowledge that the response during the treatment session of the patients included in the studies listed remains unclear, as the only information available is the final outcome. Thus, it is uncertain whether the response was gradual or occurred only after the treatment, as was the case with our patient. This delayed response to treatment may be attributable to the time needed for the body's immune system to fully develop against HPV or to the cumulative impact of multiple vaccine doses administered over an extended period. The findings of Mohtashim M et al. [6] align with our study's observations that this observed improvement cannot be credited to spontaneous resolution due to the warts being refractory in nature. Consequently, studies with shorter follow-up periods may have underestimated the efficacy of intralesional MMR therapy for warts. To address this issue, we propose that future studies be conducted with longer follow-up periods, thus allowing us to gain a more accurate understanding of the mechanism and efficacy of MMR therapy for warts.

## Conclusions

This case study highlights the complexity of treating recalcitrant verruca vulgaris and underscores the potential efficacy of MMR intralesional immunotherapy as a treatment modality. Despite initial non-responsiveness to standard topical and destructive treatments, the patient ultimately achieved complete resolution of palmoplantar and periungual warts with intralesional MMR. The delayed response observed in this case suggests that a prolonged immune response may be necessary for full therapeutic effect, warranting the need for a longer follow-up.

The case supports the use of MMR intralesional immunotherapy, particularly in patients with multiple or recalcitrant warts, due to its advantages of lower cost, fewer side effects, minimal scarring, and the potential for treating both primary and distant warts. Further research is needed to establish standardized dosing and follow-up protocols, and to better understand the immunological mechanisms driving this response. Nevertheless, the results of this case contribute to the growing body of evidence favoring MMR as a valuable alternative in the management of recalcitrant warts, offering a promising option for patients who have not responded to conventional treatments.
